# Improving the Genome Editing Efficiency of CRISPR/Cas9 in Melon and Watermelon

**DOI:** 10.3390/cells13211782

**Published:** 2024-10-28

**Authors:** Zhuanrong Wang, Lili Wan, Jian Ren, Na Zhang, Hongxia Zeng, Jiaqi Wei, Mi Tang

**Affiliations:** Institute of Crop, Wuhan Academy of Agricultural Sciences, Wuhan 430065, China; wangzhuanrong@wuhanagri.com (Z.W.); wanlili13226@163.com (L.W.);

**Keywords:** CRISPR, Pol II promoter, gene editing, *Cucumis melo* L., *Citrullus lanatus*

## Abstract

CRISPR/Cas9 is a powerful genome editing tool for trait improvement in various crops; however, enhancing mutation efficiency using CRISPR/Cas9 in watermelon and melon remains challenging. We designed four CRISPR systems with different sgRNA expression cassettes to target the phytoene desaturase (*PDS*) gene in melon. The constructed vectors were delivered to host plants using *Agrobacterium*-mediated transformation. Phenotypic and genotypic analyses of the edited melon seedlings revealed that the CRISPR systems with tRNA and Csy4 spacers driven by the Pol II-type promoter significantly improved mutation efficiency, reaching 25.20% and 42.82%, respectively. Notably, 78.95% of the mutations generated by the Csy4 system involved large-fragment deletions (LDs) between the two target sites. In watermelon, the Csy4 system achieved a *PDS* editing efficiency of 41.48%, with 71.43% of the edited seedlings showing LD between the two target sites. Sequencing analysis indicated that the edited melon seedlings exhibited heterozygous, three-allele mutation and chimeric events; the edited watermelon seedlings included 2/14 homozygous mutations. Compared to the commonly used Pol III promoter, using the Pol II promoter to drive sgRNA expression cassettes containing Csy4 showed the best improvement in CRISPR/Cas9 editing efficiency in melon; this system was also effective in watermelon.

## 1. Introduction

Cucurbitaceae crops are an economically important fruit with rich nutritional value and a delicious taste. Total melon and watermelon production exceeds 128 million metric tonnes annually in more than 100 growing countries worldwide [1]. However, breeding improvements for these crops face the challenges of long-term and complex selection processes for traits such as dwarfism, fruit ripening, sugar transporter, and sex determination, all of which are essential for enhancing fruit yield and quality [2,3,4,5,6,7]. With the development of molecular biotechnology, especially Clustered Regularly Interspaced Short Palindromic Repeats (CRISPRs)/CRISPR-associated nuclease 9 (Cas9) and high-throughput sequencing technology, traditional molecular breeding is gradually changing from “experience breeding” to “precision breeding”. Currently, CRISPR/Cas9 gene editing technology is widely applied to major crop species, oilseeds, fruits, vegetables, and other cash crops, including rice [8], tomatoes [9], grapes [10], and cotton [11]. However, the editing efficiency remains low, limiting its application in Cucurbitaceae crops. Therefore, developing gene editing tools with higher editing efficiency for the validation of gene function and molecular design breeding in cucurbit crops is necessary.

Cucurbitaceae crops, including watermelon (*Citrullus lanatus*), cucumbers (*Cucumis sativus* L.), melon (*Cucumis melo* L.), and squash (*Cucurbita moschata* D.), are significantly resistant to *Agrobacterium* in genetic transformation [12]. This resistance makes it difficult to obtain positive and stably inherited transformation lines using the *Agrobacterium*. Increasing developmental regulators and employing physical methods, such as nano-brushing and vacuum pressurisation, have significantly improved the genetic transformation efficiency in cucurbit crops [12,13,14]. These studies provide a foundation for the application of gene editing in watermelon and melon. Gene editing in melon was first reported in 2019 with an editing efficiency of 1.77% [15]. Melon ‘Védrantais’ (VED) material has the highest regeneration efficiency germplasm reported to date (approximately 12–15%), with editing efficiencies of 15%, 40%, and 46% when editing the *NON-RIPENING* (*NAC-NOR*), *CONSTITUTIVE TRIPLE RESPONSE 1* (*CRT1*), and *REPRESSOR OF SILENCING 1* (*ROS1*) genes in melon, respectively [16,17]. In watermelon, gene editing technology has been successfully used to create double haploid mutants [18] and to resolve sugar regulation and seed germination regulation mechanisms [6,19], and a single-base editing system can also work in watermelon [20]. However, the editing efficiency of melon and watermelon is still relatively low compared to the gene editing rates of rice (>80%) and maize (87%) [21,22,23,24,25].

The mutational efficiency of the CRISPR/Cas9 system is affected by a variety of factors, including the expression abundance of the Cas9 protein [26], the composition of the single guide RNA (sgRNA) cassette involving the promoter, terminator, and the target sequence spacer [25,27,28]. To enhance the efficiency of genome editing, SpCas9 (*Streptococcus pyogenes*) proteins can be modified with species-specific codon optimisation [29]. Moreover, constitutively expressed plant promoters were commonly used to boost Cas9 protein expression, such as the *Cauliflower mosaic virus* 35S (CaMV 35S) promoter, ubiquitin gene promoter (UBQ) [10,25,30,31], and *Arabidopsis* YAO (*At4g05410*) [32]. Previous studies have demonstrated that the *Arabidopsis* UBQ10 promoter-driven Cas9 significantly enhanced the mutation efficiency four-fold compared to the CaMV 35S promoter in *Arabidopsis* [30].

In the sgRNA expression, four RNA cleavage processes have been mainly used in the plant CRISPR/Cas9 system: (1) the plant RNA polymerase III (Pol III) promoter to separate individual gRNA expression cassettes, such as *Arabidopsis* AtU3 and AtU6 [33], (2) Csy-type (CRISPR system *yersinia*) ribonuclease 4 (Csy4), RNA endoribonuclease from *Pseudomonas aeruginosa*, (3) the plant endogenous transfer RNA (tRNA) processing enzyme cleavage system [28], and (4) ribozymes [34]. The Pol III promoters are widely used in various crops and also enable the expression of polycistronic tRNA-sgRNA constructs involved in multigene-targeted genome editing [28,35]. In particular, the use of the species-specific Pol III promoter increases the ability to express small RNAs and the complexity of the sgRNA expression cassette [10,36]. In the tRNA spacer CRISPR/Cas9 system, the relative expression level of sgRNA was higher than that of the sgRNA expression transcribed by a separate Pol III promoter [28,37]. However, in plants, the widespread expression of Pol III promoter-driven sgRNAs across all tissues and throughout every stage of growth and development can lead to a lack of spatial and temporal control, potentially limiting their effectiveness for certain target genes [28,34]. Pol II promoters can also be used to initiate sgRNA expression cassettes as they can transcribe longer transcripts, such as the *Arabidopsis* ribosomal protein ES8Z (*At5g20290*) promoter [38] and the *Cestrum Yellow Leaf Curling Virus* (CmYLCV) promoter [27,39]. The CmYLCV promoter-driven tRNA and Csy4 systems have been successfully used to improve editing efficiency in a variety of plants including tomato [27], sweet orange [39], and alfalfa [40]. In the Pol II CRISPR/Cas9 system, when Csy4 ribonuclease and tRNA processed polyribonucleotide sgRNA transcripts, the mutation frequency was nearly 2-fold higher than when each sgRNA was expressed by a separate Pol III promoter [27]. Further, the Csy4 system consistently produces a higher frequency of deletions in tomato protoplasts, and multiple sgRNA transcripts were shorter than the tRNA system [27].

To obtain efficient vector systems for gene editing in melon, we compared the efficiencies of the Pol II and Pol III promoters, as well as comparing Csy4 and tRNA for the expression of sgRNA cassettes in melon. Subsequently, we then investigated their genome editing efficiency by targeting the melon phytoene desaturase (*PDS*) gene. We determined whether the use of the Pol II CmYLCV promoter can improve editing efficiencies in melon and result in a higher frequency of large-fragment deletion (LD)-type mutations with Csy4. Ultimately, we established efficient gene editing vectors that could act as new tools for gene function studies in the other cucurbit germplasms.

## 2. Materials and Methods

### 2.1. Gene Clone and sgRNA Selection

The coding DNA sequences of *PDS* from melon varieties cv. Védrantais (VED) and watermelon variety (D66) were obtained from the Cucurbit Genomics Database (accession codes *MELO3C017772.2* and *Cla97C07G142100*, respectively). VED was provided by Dr. Bin Liu from Xinjiang Academy of Agricultural Sciences, and D66 was our variety, which was a yellow-fleshed small watermelon. To ensure the absence of potential genotypic single nucleotide polymorphisms that could impact genome editing due to mismatches, genomic DNA was extracted from VED and D66 using a modified method [41] (Table S1). The target region was then amplified using polymerase chain reaction (PCR) and validated through Sanger sequencing.

For *Cucumis melo* L. *PDS (CmPDS*) and *Citrullus lanatus PDS* (*ClPDS*) knockout, three sgRNAs were designed from coding sequence (CDS) regions using http://crispr.hzau.edu.cn/CRISPR2/ (accessed on 15 November 2023). The selected three sgRNAs can target the exon of both *CmPDS* and *ClPDS* at the same time, and the sequences are sgRNA1 (minus strand), 5′-TAATGGAGAACAGCATCTCGAGG-3′; sgRNA2 (minus strand), 5′-GTAGTGAGATTGTGGGCGATGGG-3′; and sgRNA3 (minus strand), 5′-TATCATCTATCTGTGGTCTAGGG-3′. To select efficient target sites, in vitro transcription and Cas9 cleavage experiments were performed on three target sites. First, the substrate DNA fragment containing three sgRNAs was amplified with PDS407F/R primers (Table S2). PCR products were recovered using the gel extraction kit (Tiangen, Beijing, China) following the manufacturer’s instructions, and the PDS-QT1-376, PDS-QT2-377, and PDS-QT3-408 primers (Table S2) were used to transcribe the three sgRNAs sites in vitro (Inovogen, Chongqing, China). The transcription sgRNAs were incubated with Cas9 protein (Inovogen, Chongqing, China) and substrate DNA fragments at 55 °C for 5 min and then detected via 2% agarose gel electrophoresis. Finally, the efficient sgRNA1 and sgRNA3 sequences were synthesised and inserted into different vectors.

### 2.2. Construction of pBM-C System CRISPR Vectors

**Figure 1:**
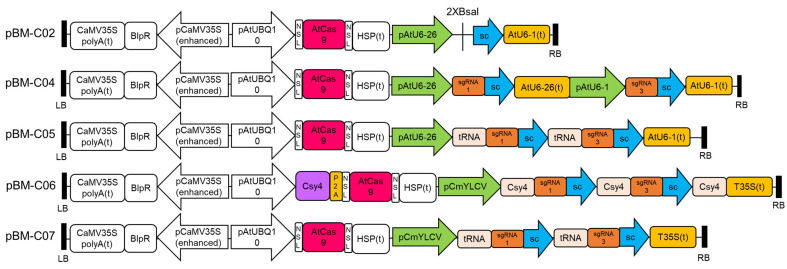
Construction of CRISPR pBM-C vectors. RB and LB represent the right and left borders of *Agrobacterium*. *BlpR*: phosphinothricin acetyltransferase, which produces bialaphos resistance (*Bar*). sc: sgRNA scaffold. (t): terminator. AtUBQ10: ubiquitin 10 gene promoter from *Arabidopsis.* NLS: Nuclear localization signal. AtCas9: *Arabidopsis* codon optimisation of Cas9. HSP (t): the heat shock protein 18.2 (HSP) terminator from *Arabidopsis*. T35S (t): *Cauliflower Mosaic Virus* 35S terminator.

To compare the editing efficiency of different vector structures, four different dual sgRNA CRISPR pBM-C vectors were constructed (Figure 1). pBM-C underwent modifications using the pYL_Cas9Pubi_B construct [24]. Initially, the synthetic sequence pAtUBQ10+SV40NLS (Table S3) was inserted between the EcoRI and DraIII enzyme sites in the pYL_Cas9Pubi_B vector. Subsequently, the sequence AtCas9+SV40NLS+HSP (terminator, t) obtained from the PMOD_A501 vector (Addgene, Plasmid #91011) was integrated between the DraIII (New England Biolabs, NEB, USA) and MauBI (Thermo Scientific, Vilnius, Lithuania) cleavage sites via homologous recombination (Vazyme, Nanjing, China). Following this, the pAtU6-26+2XBsaI+gRNA-sc+AtU6-1 (t) module was obtained from the pKSE401 vector [34] and inserted at the AscI site to create the pBM-C02 vector. With pBM-C02 as the base skeleton, pBM-C04 and pBM-C05 use the Pol III U6 promoter from *Arabidopsis* to express the sgRNA cassette, while pBM-C06 and pBM-C07 use the Pol II promoter CmYLCV to express the sgRNA cassette. In the pBM-C06 vector, the Csy4 endonuclease sequence was synthesised after pAtUBQ10, and P2A was used to connect the Csy4 endonuclease sequence and AtCas9; Csy4 was used to space different sgRNAs (Figure 1). Subsequently, the terminator of AtU6 was used for pBM-C04 and pBM-C05 vectors, while the T35S terminator was used for pBM-C06 and pBM-C07 vectors to enhance the transcription termination of the sgRNA expression cassette (Figure 1). Ultimately, highly efficient sgRNA1 and sgRNA3 were ligated into the vectors with synthetic methods, resulting in the formation of four dual-targeted gene editing vectors (Figure 1). The detailed structure of the pBM-C vectors is shown in Figure 1. The synthesised sequences are shown in Table S3.

### 2.3. Agrobacterium-Mediated Transformation of Melon and Watermelon

For the development of *Agrobacterium*-mediated transformation and genome editing systems, melon variety cv. Védrantais (VED) and watermelon variety D66 were used as the transformation receptor for *PDS* gene editing using the four created genome editing vectors and a control vector. Cotyledon explants of watermelon and melon were prepared, as reported by Wan [42]. The optical density (OD) of the prepared *Agrobacterium* containing the target vector was measured using OD_600_ = 0.1 in 20 mL of suspension [16]. For melon transformation, cotyledons were gently scratched 2–3 times with a nano-brush (KITA, Nanotek Brush NANO-3-003, Tokyo, Japan). The explants and *Agrobacterium* suspension were mixed in a conical flask, placed in an ultrasonic water bath (Dingtaihengsheng, DTC-10J, 40 KHz, Wuhan, China) for 10 s, and then placed in a vacuum (Feiyue oil-free vacuum 2FY-2C-N, Taizhou, China) at −0.8 kPa for 90 s [42]. The watermelon explants were immersed in the vector *Agrobacterium* suspension for 15 min. After co-culture for 48 h, explants were transferred onto a selective induction medium. Then, the regenerated seedlings were transferred onto selective elongation and rooting medium to obtain T0 transgenic plants. For melon transformation, the medium composition was the same as that used by Liu [16]. For the watermelon transformation, the medium composition was the same as that used by Feng [14]. All explants and regenerated seedlings were cultured under a 16 h light/8 h dark cycle at 28 °C.

To determine the efficiency of genetic transformation, we tested regenerated seedlings obtained after tissue culture for the bialaphos resistance (*Bar*) gene. The primers are shown in Table S2. Regenerated seedlings in which the *Bar* gene was detected were defined as transgene-positive seedlings. The genetic transformation efficiency was based on the total number of *Bar-*positive seedlings/total number of explants after *Agrobacterium* infiltration. The editing efficiency was the number of seedlings where editing occurs at the target site (s)/number of *Bar*-positive seedlings.

### 2.4. On- and Off-Target Efficiency Analysis

Hi-TOM is a high-throughput method for detecting target site editing genotypes in a single plant [43]. The genomic DNA was extracted from T0 individual regenerated plant tissues as previously mentioned in 2.1 (Table S1), and this DNA was used as the template for PCR amplification. The PCR product library was established through two rounds of PCR. The first round of PCR used specific primers with bridging sequences of 5′-GGAGTGAGTACGGTGTGC-3′ for the forward primer and 5′-GAGTTGGATGCTGGATGG-3′ for the reverse primer at the 5′ end (Table S2) to amplify the fragments containing the sgRNA1and sgRNA3 sequence. PCR was carried out in a 10 μL reaction volume that included 0.5 μM of each primer, 5.0 μL of 2× Taq Master Mix (Vazyme, Nanjing, China), and 50 ng of DNA template. PCR conditions consisted of 33 amplification cycles (94 °C for 30 s, 56 °C for 30 s, and 72 °C for 30 s) in total. For the second round of PCR, 1 μL of the first round PCR product was diluted 10× as a template, and each sample was amplified with 4 bp barcode tags added to the 5′ end of the primers to distinguish between different samples. After forming the PCR product library, samples were purified (Tiangen, Beijing, China), sequenced on an Illumina platform (~1 G data), and analysed via the Hi-TOM 2.0 website with a 5% filter threshold [43].

For off-target analysis, CRISPR-P v 2.0 (hzau.edu.cn, accessed on 15 November 2023) was used to identify potential sites. Five edited seedlings were selected for off-target site detection. PCR amplification of the corresponding off-target sites was performed using the primers listed in Table S2. After TA cloning, the off-target sites were analysed using Sanger sequencing.

### 2.5. Statistical Analysis

The tissue culture experiment was performed using three biological replicates to determine editing efficiency. According to the results of the Hi-TOM 2.0 website analysis [38], the editing types of each plant and the statistics of editing efficiency were carried out using GraphPad 6. The mean value was used for statistical analysis, and statistical significance was assessed according to the one-way analysis of variance (ANOVA), where the significance level of *p* < 0.05 was considered statistically significant.

## 3. Results

### 3.1. Screening Efficient sgRNA In Vitro

**Figure 2:**
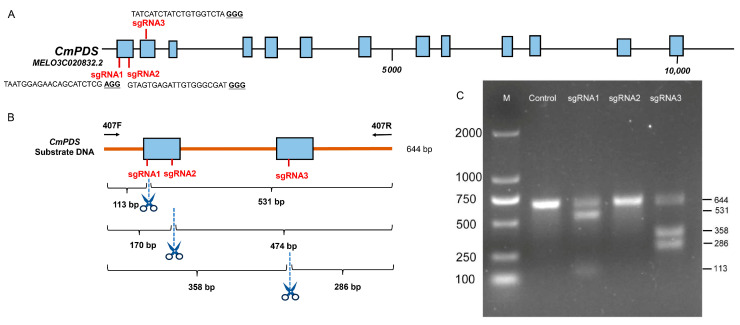
Selection of highly efficient sgRNAs and construction of CRISPR vector for melon transformation. (**A**) Structure of the target gene *CmPDS*. The blue boxes represent exons. The three selected sgRNAs are located in the first and second exons. (**B**) Schematic diagram of sgRNA cleaving the substrate DNA. (**C**) Efficiency of different sgRNAs in cleaving substrate DNA in vitro. M: DL2000 marker. Control: the substrate DNA. Three sgRNA wells contain Cas9, transcripts of different sgRNAs, and substrate DNA.

To screen the efficiently available sgRNAs from the predicted website, we selected three sgRNAs targeting the first and second exons of the *CmPDS* gene (Figure 2A); they all had a low predicted off-target rate. We obtained high levels of transcripts of these three sgRNAs through in vitro transcription and performed enzyme digestion experiments on PCR substrate DNA containing sgRNA target sequences using spCas9 protein and sgRNA transcripts to screen for efficient target sgRNAs. When the sgRNA is effective, it cleaves the substrate DNA at the corresponding positions, resulting in two DNA fragments of different sizes. Theoretically, sgRNA1 can cleave the substrate DNA into fragments of 113 and 531 bp; sgRNA2 can cleave the substrate DNA into fragments of 170 and 474 bp; and sgRNA3 can cleave the substrate DNA into fragments of 358 and 286 bp (Figure 2B). Agarose gel electrophoresis showed that sgRNA1 and sgRNA3 could efficiently cleave 644 bp substrate DNA. Specifically, sgRNA1 produced fragments of 113 bp and 531 bp, while sgRNA3 produced fragments of 358 bp and 286 bp (Figure 2C). In contrast, sgRNA2 did not effectively cleave the 644 bp substrate DNA (Figure 2C). These results indicated that they were high-efficiency sgRNAs. Therefore, we selected sgRNA1 and sgRNA3 as efficient targets for addition to the four designed CRISPR system vectors (see Figure 1).

### 3.2. PDS Mutants Phenotypic and Comparison of pBM-C Vector Editing Efficiency

**Figure 3:**
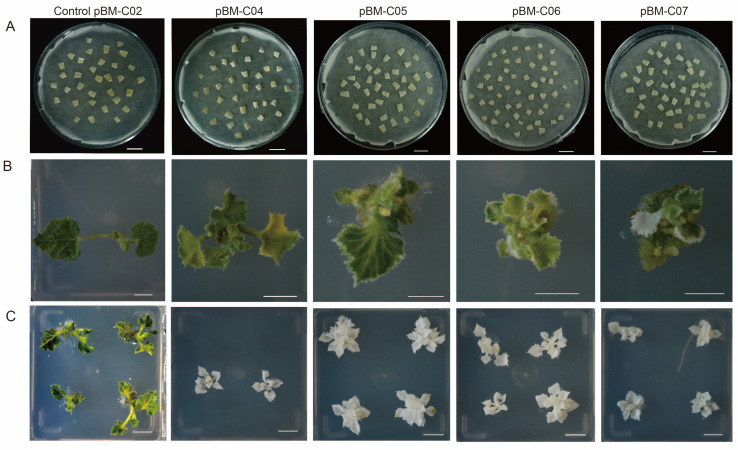
Phenotypes among four pBM-C CRISPR vectors. The pBM-C02 is the control vector. (**A**) The explants after immersion with different *Agrobacterium* edit vectors following physical treatment. Bar = 1 cm. (**B**) The chimeric phenotypes of melon seedlings are mainly characterised by leaf edge albinism. Bar = 1 cm. (**C**) The completely albino phenotypes with different vectors. Bar = 1 cm.

To test the applicability of the four pBM-C system vectors in melon, we used *Agrobacterium*-mediated genetic transformation to transfer four vectors with two highly efficient sgRNAs into melon VED cultivars (Figure 3A). The pBM-C02 vector (without sgRNA) was used as a control for the simultaneous transformation of melons. To improve transformation efficiency, the addition of *Agrobacterium* to explants followed by treatment with micro-brushing, sonication, and vacuum infiltration treatment. The results showed that the range of transformation efficiency for VED was 30.58 to 42.3% (Table 1). Compared to the control vectors, all four pBM-C systems produced albino, chimeric, and wild-type regeneration seedlings (Figure 3B,C). For the pBM-C04 vector, 2 chimeric and 2 fully albino seedlings were obtained from 46 *Bar*-positive seedlings, while the pBM-C05 vector produced 2 chimeric and 4 fully albino seedlings from 51 *Bar*-positive seedlings (Table 1). In the case of the pBM-C06 vector, 7 chimeric and 8 fully albino seedlings emerged from 44 *Bar*-positive seedlings, and the pBM-C07 vector resulted in 3 chimeric and 4 fully albino seedlings out of 43 *Bar*-positive seedlings (Table 1). For further accurate statistics on edit rates, we performed Hi-TOM sequencing to further validate the mutation efficiency of each vector. Plants with a target editing ratio greater than 5% (number of reads with the target mutation divided by the total number of reads at the target locus) were considered “edited” plants. The results showed that the editing efficiencies of the different vectors were 8.62% (pBM-C04), 15.77% (pBM-C05), 42.82% (pBM-C06), and 25.2% (pBM-C07), respectively (Table 1). Statistical analysis of editing efficiencies revealed that the editing efficiency of the pBM-C06 vector was significantly higher than the pBM-C04 and pBM-C05 vectors (*p* < 0.05). Although there was a large numerical difference in editing efficiency between the pBM-C06 and pBM-C07 vectors, statistical analysis indicated that this difference was not significant (Table 1). These results suggested that the pBM-C06 (Pol II-Csy4-type) system had the highest editing efficiency, followed by the pBM-C07 (Pol II-tRNA-type) system.

### 3.3. Comparison of the Types of Edits Produced by Different pBM-C Vectors

**Figure 4:**
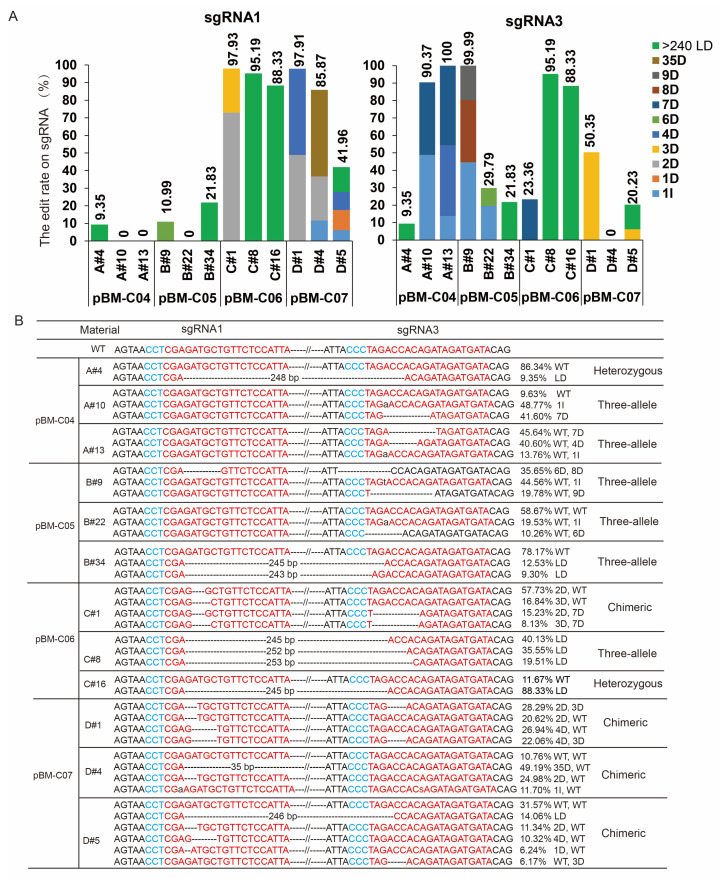
Editing types among different pBM-C CRISPR vectors. (**A**) The stacked column diagram showed the target mutation rate based on Hi-TOM analysis (reads of target mutation/total reads of the target site) for representative mutations. A total of mutation types > 95% indicated complete mutations at the sgRNA sites. Different colours represented different mutation types. D indicated deletion, I indicated insertion, and LD indicated large-fragment deletions between sgRNA1 and sgRNA3. (**B**) The specific editing genotypes of different vectors at the two sgRNA sites. The numbers on the right represented the proportion of the current editing type at two sgRNA sites based on Hi-TOM analysis.

Due to the varying specificity of different target sites, different sgRNA sites exhibit different editing efficiencies. To analyse the editing types at the two target sites, we statistically evaluated the editing results of different vectors. Hi-TOM results showed that the number of edits at sgRNA3 using the pBM-C04 and pBM-C05 vectors was four times higher than that at sgRNA1, while the number of edits at sgRNA1 using the pBM-C07 vector was higher than those at sgRNA3 (Table 2). The number of mutations at the two target sites using the pBM-C06 vector was similar, mainly because the proportion of LD between the two target sites in pBM-C06 accounted for 15/19 (Table 2). Further analysis revealed that the proportion of LD generated by the pBM-C06 vector reached 78.95%, which is 2.3 to 6.3 times higher than that of other vectors producing LD-type mutations (Table 2). Hi-TOM analysis showed that four CRISPR pBM-C vectors generated mutants containing 1 bp insertions, indels, and LDs (Figure 4A,B). Specifically, the mutations induced by the pBM-C04, pBM-C05, and pBM-C07 vectors predominantly involved 1 bp insertions and <10 bp indels (Figure 4A,B). Complete editing of a single sgRNA site was present in edited seedlings produced by all four vectors. (the total of the mutation types > 95%, Figure 4A). Analysis of the two sites simultaneously showed that all edited seedlings contained chimeras, heterozygotes, and three alleles (Figure 4B). These results suggest that the Pol II vector pBM-C06 had a greater advantage in generating large deletions between the two neighbouring sites compared to other vectors.

### 3.4. The Off-Target Effect Analysis in Melon

It is necessary to detect off-target problems because off-targeting may cause other unintended results or negatively affect plant growth and development. In this study, we predicted the potential off-target sites for sgRNA1 and sgRNA3. Although no off-target risks were identified for sgRNA1 and sgRNA3 using Cas-OFFinder, CRISPR-P 2.0 indicated that sgRNA1 has 10 potential off-target sites and sgRNA3 has 9 potential off-target sites (Table 3). We analysed the top five potential off-target sites with the highest scores for each and found that three of sgRNA1’s and two of sgRNA3’s potential off-target sites are located within CDS gene regions. Sanger sequencing results revealed that the pBM-C06 vector did not induce mutations at these potential off-target sites (Figure S2). The above results indicated that the selected sgRNA1 and sgRNA3 are specific in editing the melon *PDS* gene without off-targeting.

### 3.5. The pBM-C06 Vector Achieves Efficient Editing in Watermelon

**Figure 5:**
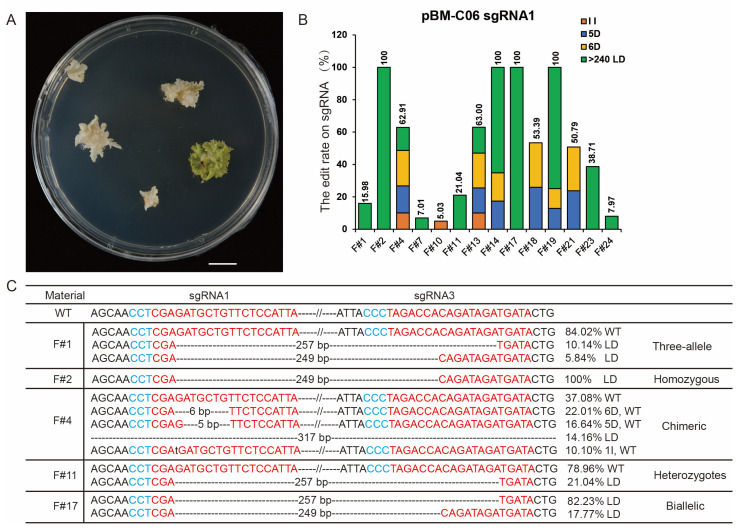
Phenotypes and editing types in watermelon using the pBM-C06 vector. (**A**) Albino and chimeric seedlings of watermelon on selection medium. (**B**) The columnar stacking diagram shows the target mutation rate based on Hi-TOM analysis. The pBM-C06 vector exhibits four types of edits at sgRNA1 and sgRNA3: 1-base insertion, 5- or 6-base deletion, and > 240 bp large indels between the two targets. The sgRNA3 site has no other type of editing except for large deletions. (**C**) Specific editing genotypes at sgRNA sites for five representative mutations. The numbers on the right represent the proportion of the current editing type based on Hi-TOM analysis.

Since the pBM-C06 vector exhibited the highest genetic transformation efficiency in melon, and sgRNA1 and sgRNA3 effectively targeted the watermelon *ClPDS* gene (Figure S1), we performed genetic transformation using the pBM-C06 vector in the watermelon D66 cultivar. Due to the high contamination rate observed after treating watermelon explants with physical methods involving nano-brushes, ultrasound, and vacuum pumps, the watermelon explants were immersed in the pBM-C06 *Agrobacterium* suspension for 15 min. We obtained 34 *Bar*-positive seedlings from 221 cotyledon explants, which showed a transformation efficiency of 15.44%. In watermelon, the pBM-C06 vector was highly efficient in producing regeneration seedlings with albino and chimeric phenotypes (Figure 5A), and Hi-TOM results showed that the gene editing efficiency was 41.48% (14/34). Genotypic analysis of 14 edited seedlings revealed four types of resulting mutations: 1-base insertion, 5- or 6-base deletion, and >240 bp LD between the two target sites, while the sgRNA1 site had no other type of editing except for large deletions (Figure 5B,C). The LD-type mutation between the two target sites accounted for 71.43% (10/14) of all mutations. The pBM-C06 vector produced 14 edited seedlings containing homozygous, heterozygotes, chimeras, biallelic, and three alleles (Figure 5B,C), four of which had 100% mutation frequency at the sgRNA1 site (Figure 5B). These results suggest that the Pol II vector pBM-C06 is also suitable for watermelon genome editing and is also more likely to produce large deletions between the two neighbouring sites.

### 3.6. Comparison of the Time Appearance of Albino Phenotypes

During genetic transformation in melon, the pBM-C04 vector required 59 days before albino shoots were first observed, whereas albino phenotypes were observed with other vectors at approximately 35 days (Table 3). These results showed that the pBM-C05, pBM-C06, and pBM-C07 vectors displayed albino phenotypes 14 days earlier than the pBM-C04 vector on the selection medium. The results of the significance analysis indicated that this 14-day difference was statistically significant (*p* < 0.05). Additionally, the pBM-C06 vector demonstrated a significant advantage in rapidly generating edited watermelon seedlings, as the albino phenotype was observed within 32 days of the genetic transformation process (Table 4). These results indicated that the efficient editing system has the advantage of rapidly obtaining mutant regenerated seedlings in watermelon and melon.

## 4. Discussion

The CRISPR/Cas9 system holds great potential for studying plant gene function, as it can generate single or multiple mutations without introducing foreign inserts. However, in cucurbit crops, its application faces several bottlenecks due to complex factors, such as low genetic transformation efficiency, high chimeric gene editing rates, and difficulty obtaining mutants with stable inheritance [34]. In this study, we used a combined physical method, including ultrasound, nano-brushing, and vacuum infiltration [12] to increase the transformation efficiency of VED to 30.58–42.30%, which is more than twice as high as the results of previous studies [16]. Notably, this transformation efficiency is equivalent to the level achieved by adding developmental regulator factors to the vector to improve transformation efficiency [42]. However, when we used the combined physical method for watermelon genetic transformation, it did not significantly improve the transformation efficiency but rather led to excessive bacterial overgrowth. This may be because watermelon requires a lower bacterial OD value when using this method. Additionally, watermelon explants may be more sensitive to injury; therefore, a soaking inoculation method may be better for improving transformation efficiency.

Software-predicted sgRNAs often fail to induce cleavage at the target site [44], necessitating a different approach for detecting sgRNA efficiency. The protoplast transformation method has been used to test the gene editing efficiency of sgRNA in watermelon [45]; however, it is limited in its applicability to other plants since the transformation of protoplasts is difficult to succeed or with low efficiency [44]. Recent studies have shown that efficiency can be detected using the *Agrobacterium*-mediated hairy root transformation protocol [46]. Additionally, studies have assessed the efficiency of selected sgRNAs by measuring the editing efficiency of calli after transformation [47]. These methods take a long time to confirm the effects of sgRNA; therefore, we chose to transcribe the selected sgRNA in vitro and use commercial Cas9 protein to cleave the target substrate DNA and detect the sgRNA efficiency. With this method, the tissue culture process, or protoplasts, is avoided, and the efficiency of sgRNA can be confirmed in just 3–5 days. However, this in vitro cleavage assay, without interference from the background genome, may also produce false positives. Therefore, detecting regenerating shoots in the screening media is necessary to further test the efficiency of sgRNA. The time of emergence of albino seedlings found in this study also provides a reference time for detecting sgRNA efficiency using regenerating shoots. During the genetic transformation of watermelon and melon, the albino phenotype appeared at around 32–35 days when using tRNA and Csy4 vectors (Table 4). Thus, we recommend sampling regenerated shoots at 50–60 days (counting from germination) to test sgRNA efficiency, as this avoids having too few edited buds at the early stage.

The codon optimization of Cas9 [26], the promoter of Cas9 expression [30], and the arrangement of sgRNA expression cassettes [25,27,28] may affect the expression level and targeting efficiency of Cas9. The pBSE401 and pHSE401 systems are driven by the CaMV 35S promoter with a plant-codon-optimized spCas9 vector [33]. In the early stages of this study, we did not obtain gene-edited melon seedlings with this pBSE401 system (Pol III promoter vector), even though the positive transgenic rate reached about 30%. However, pBSE401 produces efficient editing in watermelon [19,48]. The pHSE401 vector produces editing events normally in both watermelon and melon, but the editing rate is low [15,45]. The pB7-CAS9-TPC vector had the highest editing rate (15–46%) reported so far in melon, but the details of the vector were not reported [16]. We speculated that this result may be related to the expression levels of Cas9 and the sgRNA cassette [25,27,28,30]. Previous research has indicated that using plant constitutive expression promoters results in a higher edit rate than the CaMV 35S promoter [40]. We replaced the commonly used CaMV 35S promoter with the AtUBQ10 promoter to drive an *Arabidopsis*-codon-optimized Cas9. Building on this modification, the editing rates of from 8.62 to 42.82% were observed with the four different vectors, suggesting that the AtUBQ10 system is more effective than the CaMV 35S system.

Moreover, different methods of spacing sgRNA cassettes have been shown to have varying impacts on the editing rate [27,28]. In this study, the four tested vectors targeting the same targets produced different editing efficiencies. Notably, the tRNA and Csy4 systems driven by the Pol II-type promoter (CmYLCV) exhibited higher gene editing frequencies, which is consistent with results observed in rice and *Arabidopsis* [27,28]. The results demonstrate that using tRNA-spaced sgRNA expression cassettes under the Pol II-type promoters can significantly enhance gene editing efficiency, with a 2.9-fold increase compared to the Pol III promoters (Table 2, pBM-C07 vs. pBM-C04). Furthermore, using the Pol II promoter with Csy4-spaced sgRNA cassettes can achieve a 5.0-fold increase in editing rates compared to Pol III promoters (Table 2, pBM-C06 vs. pBM-C04). For the Pol III promoter, the express sgRNAs cassette needs to recognise specific initial nucleotides [49]. The U3 and U6 promoters have distinct transcription start sites, with adenine (A) and guanine (G), respectively [35]. These could decrease the effectiveness of targeted editing, but the Pol II promoter does not have this limitation. The enhanced efficiency is likely due to the higher abundance of expression driven by Pol II-type promoters, which is not hindered by internal small unit termination sites and produces long transcripts, thereby increasing the overall editing efficiency [27].

In the CRISPR/Cas9 system, when the target DNA is cut by the Cas9 protein to form a double-strand break, the non-homologous end-joining repair pathway is preferentially initiated in the recipient cells. This process typically generates base insertion or deletion mutations (Indels) 3 bp upstream of the protospacer adjacent motif site, with most mutations involving a 1 bp insertion and smaller short fragment deletions [25,50]. In this study, we found that the Csy4 system was able to produce higher frequencies of large-fragment deletions (>240 bp), which is consistent with results observed in *Arabidopsis* [27,51]. By designing a sgRNA site on each side of the target gene segment, chromosome fragments larger than 100 kb can be successfully deleted, and smaller fragments of less than 1 kb (especially <100 bp) can be deleted with higher efficiency [52,53,54]. All pBM-C vectors obtained very low percentages of homozygous mutants in melon and watermelon. These results suggested that the method of genetic transformation of somatic embryogenesis is more prone to the presence of chimeric phenotypes, as observed in soybeans and apples [55,56]. Nevertheless, the number of chimeric genotypes can be effectively improved by increasing transformation efficiency and optimising gene editing tools. For example, the use of the Csy4 type vector increased the proportion of mutations with 2–3 editing genotypes (Figure 4A and Figure 5B). Further, a 10/14 ratio of pure LDs was observed in edited watermelon seedlings, indicating a significant efficiency improvement of the editing tool. Although we did not test the LD efficiency of distant dual-target sgRNAs and the editing efficiency of different genes, the results of this study showed that the combination of the Pol II promoter with the Csy4/tRNA system not only improves the editing efficiency but also results in a higher frequency of large-fragment deletion events. These would be very suitable for materials that are difficult to transform and where the genetic transformation cycle is too long.

Currently, the application of CRISPR/Cas9 in melon and watermelon is mainly focused on single gene loss of function mutations. Here, the two Pol II-type promoter-mediated tRNA and Csy4 vectors were not only highly efficient for editing but could also be used to achieve multigene knockout. Based on related research methods, these vectors could be used to assemble at least six sgRNAs in tandem, according to the Golden Gate method [57]. To achieve multiplex gene knockout, all sgRNAs were organised into a tandem array by tRNA or Csy4 spacers and transcribed from the Pol II-type promoter into a single transcript, and that single transcript underwent post-transcriptional processing to excise individual sgRNAs [25,27,28]. In addition, the sequences of the sgRNA expression cassettes by the tRNA and Csy4 were shorter (e.g., tRNA, 77 bp pre-tRNA^Gly^ genes; Csy4, 20 bp Csy4 hairpin) than those expressed by the Pol III promoter, suggesting that the tRNA and Csy4 system were simpler and more effective for targeted genome modification [27,28,37]. One recent study has effectively increased the efficiency of primer editing (PE) with the CmYLCV promoter-driven Csy4 structure in wheat [58]. Therefore, adding the required components for the base editor or PE vectors based on the pBM-C06 backbone could help to expand the gene editing tools for cucurbit crops.

## 5. Conclusions

In summary, we have established a highly efficient CRISPR/Cas9 system for genome editing in melon and watermelon. We confirmed that our pBM-C06 vector can achieve a high editing efficiency of 42.82%. Furthermore, this study successfully applied the system to gene loss-of-function studies by designing two sgRNAs with an interval of > 240 bp for large-fragment deletion, resulting in fragment deletion rates of 78.95% and 71.43% in mutant melon and watermelon, respectively. Overall, the highly efficient pBM-C06 vector can be used not only for single gene knockout but also has significant potential for application in multiple gene knockouts. This could expedite functional genomics research for breeding and improvement in cucurbit crops.

## Figures and Tables

**Table 1:** Transformation and editing efficiency of melon VED.

Vector	No. of Explants ^1^	No. of *Bar* Positive Seedlings ^2^	Transformation Efficiency (%) ^3^	No. of Edit T0 Plants	Editing Rate ^4^ (%)
pBM-C02 (Control)	108	35	32.46 ± 0.04	0	0
pBM-C04	129	46	35.49 ± 0.01	4	8.62 ± 0.01 ^d^
pBM-C05	122	51	42.30 ± 0.03	8	15.77 ± 0.01 ^be^
pBM-C06	144	44	30.58 ± 0.04	19	42.82 ± 0.04 ^ac^
pBM-C07	132	43	33.46 ± 0.08	10	25.20 ± 0.05 ^ab^

Data for transformation efficiency are means ± SEM of three independent trials. 1. No. of explants is the sum of explants that survived after infection in three independent trials. 2. *Bar*-positive seedlings: regenerated seedlings were detected with the bialaphos resistance (*Bar*) gene. 3. Transformation efficiency is evaluated by No. of basta-positive seedlings/No. of explants. 4. Editing rate: No. of edit T0 plants/No. of explants. Different letters (a, b, c, d, and e) indicate significant differences among treatments (*p* < 0.05).

**Table 2:** Editing efficiency of melon VED on sgRNA1 and sgRNA3.

Vector	No. of Editing T0 Plants	No. of sgRNA1	No. of sgRNA3	No. of Both sgRNAs	No. of LD	LD Rate (%) ^1^
pBM-C04	4	1	4	1	1	25.00
pBM-C05	8	2	8	2	1	12.50
pBM-C06	19	19	17	16	15	78.95
pBM-C07	10	10	4	4	2	20.00

1. LD rate: No. of LD/No. of edit T0 plants.

**Table 3:** The top five predicted off-target sites for sgRNA1 and sgRNA3.

	The Sequence of sgRNA	No. of Off-Target Sites	The Sequence of Offtarget	Off-Score	Locus	Gene ID	Region
sgRNA1	**TAATGGAGAACAGCATCTCG AGG**	10	**AAACGGATAAAAGCATCTCGCGG**	0.303	chr2:23796553..23796531		Intergenic
			**TGAGGGTGAACAGCATCACGTGG**	0.146	chr1:450775..450797	MELO3C018462	CDS
			**TAATCCAGGACAGCATCTCCAAG**	0.04	chr2:15161470..15161492	MELO3C010254	CDS
			**AAATGGAGAACAGAATCTAGGAG**	0.039	chr5:1316321..1316299	MELO3C014566	CDS
			**TCATGGAGACAAGCCTCTCGAAG**	0.014	chr6:16140751..16140729		Intergenic
sgRNA3	**TATCATCTATCTGTGGTCTA GGG**	9	** TAACATCTGTATGTGATCTAGGG**	0.357	chr11:1450252..1450230	MELO3C023325	CDS
			**TATGAACTATCAGTGGTATATGG**	0.187	chr5:27838535..27838557		Intergenic
			**TATCATCGATATGTTGCCTATGG**	0.055	chr10:21682722..21682744		Intergenic
			**TATCATCTATCTTTGATAAATGG**	0.046	chr1:20418410..20418432		Intergenic
			**TATCATCTATCAGTTATCAAAGG**	0.033	chr9:6825019..6825041	MELO3C002879	CDS

Mismatching bases are shown in red; the protospacer adjacent motif (PAM) motifs are shown in green. CDS is coding sequences.

**Table 4:** The time of appearance of albino buds in different vectors.

Vector Number	*Agrobacterium*	Receptor	Time from Germination to the First Appearance of Albino Buds (D)
pBM-C04	*EHA105*	VED	59 ± 2 ^a^
pBM-C05	*EHA105*	VED	34 ± 1 ^b^
pBM-C06	*EHA105*	VED	35 ± 1 ^b^
pBM-C07	*EHA105*	VED	35 ± 2.5 ^b^
pBM-C06	*EHA105*	D66	32 ± 1.5 ^b^

Data for transformation efficiency are means ± STDEV of three independent trials. Different letters (a and b) indicate significant differences among treatments (*p* < 0.05).

## Data Availability

The data that supports the findings of this study are available on request from the corresponding author.

## Supplementary Materials

The following supporting information can be downloaded at: https://www.mdpi.com/article/10.3390/cells13211782/s1, Figure S1. *ClPDS* gene structure map and sgRNA sites. Figure S2. Sequencing analysis of potential off-target sites in T0 edited seedlings. Table S1. Tissue DNA extraction methods. Table S2. Primers used for vector construction and transgenic plant detection. Table S3. Synthesised gene sequences.
