# Supplementary material for: Improving the Genome Editing Efficiency of CRISPR/Cas9 in Melon and Watermelon
Source: Cells. 2024 Oct 28;13(21):1782. doi: 10.3390/cells13211782 (PMC11544962; doi:10.3390/cells13211782)
Supplement: Supplementary file 1 [file cells-13-01782-s001.zip › cells-3220862-supplementary.pdf]

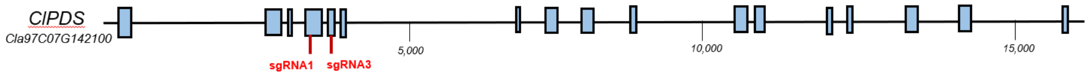

Figure S1. ***ClPDS*** **gene structure map and sgRNA sites**

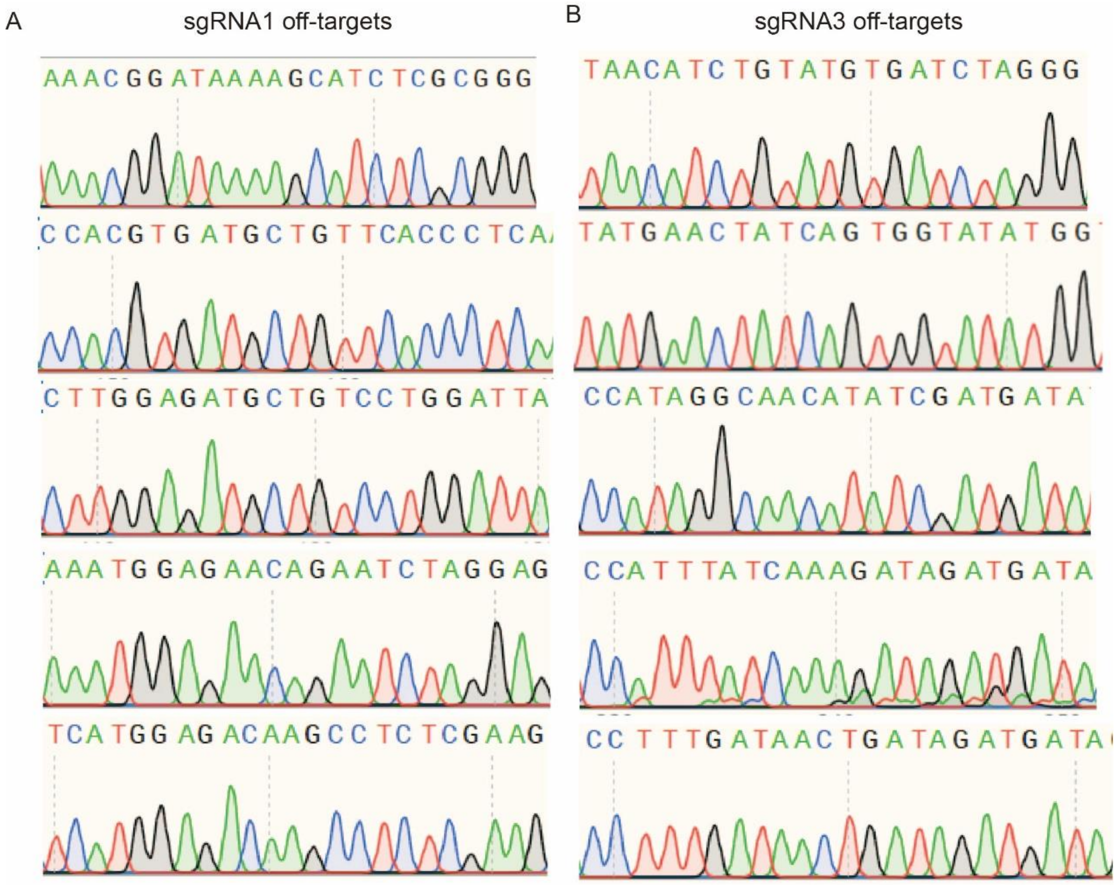

Figure S2. Prediction of potential off-target sites and sequencing analysis in T0 edited seedlings with the pBM-C06 vector. The five potential off-target sites were validated by sequencing in five different T0 edited seedlings. (A) Detection of potential off-target sites for sgRNA1. (B) Detection of potential off-target sites for sgRNA3.

Table S1. Tissue DNA extraction methods.

1	A 1 cm^2 tissue sample was disrupted with steel beads (specify model and manufacturer of grinding machine) in a 2.0 mL EP tube at room temperature with 100 μL extraction buffer (12.1 g/L Tris, 28.1 g/L NaCl, and 18.6 g/L EDTA) for 45 seconds.
2	After removing the steel beads, an additional 150 μL of extraction buffer was added, and the mixture was centrifuged at 4,000 rpm for 20 minutes.
3	75 μL of the supernatant was transferred to a fresh PCR plate, followed by the addition of 150 μL of ice-cold anhydrous ethanol. The mixture was incubated at -20°C for approximately 30 minutes and then centrifuged at 4,000 rpm for 10 minutes.
4	The ethanol was discarded, and 200 μL of 75% ethanol was added to the pellet for washing 2 minutes, followed by ethanol removal.
5	The pellet was air-dried overnight, then resuspended in 100 μL of ddH2O. After incubating at

	37°C for 1.5 hours, 1.5 μL of the DNA solution was directly used for PCR in a 10 μL reaction volume.

Tissue DNA was extracted using a modified method (Alonso et al., 2015).

Note: For watermelon and melon, the method is suitable for true leaf tissues and not for cotyledon tissues.

Table S2. List of primer pairs and synthetic sequence in this study.

Primer	Sequence (5'-3')
**Primers amplified for AtCas9 + SV40NLS + HSP(t) constructs**	
321L	CGGAAGGTTGGTATTCACGGGGTGatggataagaagtactctat
321R	TAACATAGATGACACCGCGCGCGcttatctttaatcatattcc
322F	CGGGAGCACCGGTAAGGCGCGCCGAATGATTAGGCATCGAACC
322R	CTGAGACCCTCTCTCGGTCTCCCAATCACTACTTC
323F	GAGAGAGGGTCTCAGTTTTAGAGCTAGAAATAGCA
323R	GCCGCTAGCTCGAGAGGCGCGCCTATTGGTTTATCTCATCGGA
**Primers for detect the efficient sgRNAs sites**	
PDS407F	GTTGTGGGAAGTGGGGTTT
PDS407R	AGGTTTGTGGCCAGCATCTG
PDS-QT1-376	TTAATACGACTCACTATAGG TAATGGAGAACAGCATCTCG<br>GTTTTAGAGCTAGAAATA
PDS-QT2-377	TTAATACGACTCACTATAGG GTAGTGAGATTGTGGGCGAT<br>GTTTTAGAGCTAGAAATA
PDS-QT3-408	TTAATACGACTCACTATAGG TATCATCTATCTGTGGTCTA<br>GTTTTAGAGCTAGAAATA
Bar-F	CATCGAGACAAGCACGGTCA
Bar-r	AAACCCACGTCATGCCAGTT
**Primers amplified for off-targets testing**	
476F	GTTCGGTCTGTTTGCCACTG
476R	GGGGTTCAATTCAACTCCTC
477F	GCGTCACCCCATTGAAGTAC

477R	CTGACCATGATTGCCTGTTC
478F	CGGCAACATTGGCAAAAGCC
478R	GGAGGTTGTTACCAGGTACG
479F	CGGTGAATTAAGGGGTGAGC
479R	CACCCCCACCCTTACTTATC
480F	AGAAGGTGTTCCGAGATGGG
480R	ACTCGTGCCTATGACATGGA
481F	ATTTGGCTCCACTCACTTCC
481R	TCATGGCAGCCTTGTACATG
482F	GCGAAAGAGAGTGATAGATG
482R	GGTGGAGGAAATCTGAGACC
483F	CGTGGAACCTATAGAGATAC
483R	TGGCACTTCCTTCCCACTGA
484F	GCTCTCGATTTGGTTCTAAG
484R	GACTGAAGACTTGGAAGATC
485F	CAAGGGGTTAGTTTCTTAGG
485R	GAAGGCCAATGAGATGGCTG
**Primers amplified for Hi-TOM to detect gene editing seedlings**	
CmPDS-HI-TOM-429F	ggagtgagtacggtgtgc CTGCTCTGAACTTGAGGTGG
CmPDS-HI-TOM-429R	GAGTTGGATGCTGGATGG ACTAGCAGATAAGGAAGCTG
ClPDS-HI-TOM-486F	ggagtgagtacggtgtgc CTATGTGGGTCTGTCTCTGC
ClPDS-HI-TOM-486R	GAGTTGGATGCTGGATGG GGTTTACTGGGACGTGCAGA

Table S3. Synthesised gene sequences

Synthetic sequence in the pBM-C system vector used for construction of CRISPR/Cas9 [pAtUBQ10+SV40NLS]:

agtctagctcaacagagcttttaacccaaattggtacaatagaatacaactttagatcataattctcaaaagaaagagattccttagctattctatctgc
cactccatttccttctcggcttgtatgcacaagcataaaatcctcaaacttgctaagtagatactttatgtcttggataattggattgagacttgacaagc
ataactttcatgtaaccaaagacacaagttgctgagaatccacctcaaaaatgatcttcctataattgaatcgggataatgacagcacagcccatct
aagagcctccacttctacttccagcacgcttcttacttttaccacagctcttgcacctaaccataacaccttccctgtatgatcgcgaagcacccaccc
taagccacattttaatccttctgttggccatgccccatcaaagttgcacttaacccaagattgtggtggagcttcccatgtttctcgtctgtcccgacggt
gttgtggttggtgctttccttacattctgagcctctttccttctaatccactcatctgcatcttcttgtgtccttactaatacctcattggttccaaattccctccctt
taagcaccagctcgtttctgttcttccacagcctcccaagtatccaagggactaaagcctccacattcttcagatcaggatattcttgtttaagatgttga
actctatggaggtttgtatgaactgatgatctaggaccggataagttcccttcttcatagcgaacttattcaaagaatgttttgtgtatcattcttgttacatt

gttattaatgaaaaaatattattggtcattggactgaacacgagtgttaaatatggaccaggccccaaataagatccattgatatatgaattaaataa
caagaataaatcgagtcaccaaaccacttgcctttttaacgagacttgttcaccaacttgatacaaaagtcattatcctatgcaaatcaataatcata
caaaaatatccaataacactaaaaaattaaaagaaatggataatttcacaatatgttatacgataaagaagttacttttccaagaaattcactgatttt
ataagcccacttgcattagataaatggcaaaaaaaaacaaaaaggaaaagaaataaagcacgaagaattctagaaaatacgaaatacgctt
caatgcagtgggacccacggttcaattattgccaattttcagctccaccgtatatttaaaaaataaaacgataatgctaaaaaaatataaatcgtaa
cgatcgttaaatctcaacggctggatcttatgacgaccgttagaaattgtggttgtcgacgagtcagtaataaacggcgtcaaagtggttgcagccg
gcacacacgagtcgtgtttatcaactcaaagcacaaatacttttcctcaacctaaaaataaggcaattagccaaaaacaactttgcgtgtaaacaa
cgctcaatacacgtgtcattttattattagctattgcttcaccgccttagctttctcgtgacctagtcgtcctcgtcttttcttcttcttcttctataaaacaatacc
caaagagctcttcttcttcacaattcagatttcaatttctcaaaatcttaaaaactttctctcaattctctctaccgtgatcaaggtaaatttctgtgttccttatt
ctctcaaaatcttcgattttgttttcgttcgatcccaatttcgtatatgttctttggtttagattctgttaatcttagatcgaagacgattttctgggtttgatcgtta
gatatcatcttaattctcgattagggtttcatagatatcatccgatttgttcaaataatttgagttttgtcgaataattactcttcgatttgtgatttctatctagat
ctggtgttagtttctagtttgtgcgatcgaatttgtcgattaatctgagtttttctgattaacagcctaagaagaagcggaaggttggtattcacggggtg

[Target1+gRNA-sc+AtU6-29(t)] + [AtU6-1+Target2]:

taatggagaacagcatctcggttttagagctagaaatagcaagttaaaataaggctagtccgttatcaacttgaaaaagtggcaccgagtcggtgc
ttttttttgcaaaattttccagatcgatttcttcttcctctgttcttcggcgttcaatttctggggttttctcttcgttttctgtaactgaaacctaaaatttgacctaa
aaaaaatctcaaataatatgattcagtggttttgtacttttcagttagttgagttttgcagttccgatgagataaaccaatattaatccaaactactgcagc
ctgacagacaaatgaggatgcaaacaattttaaagtttatctaacgctagctgttttgtttcttctctctggtgcaccaacgacggcgttttctcaatcata
aagaggcttgttttacttaaggccaataatgttgatggatcgaaagaagagggcttttaataaacgagcccgtttaagctgtaaacgatgtcaaaaa
catcccacatcgttcagttgaaaatagaagctctgtttatatattggtagagtcgactaagagattgtatcatctatctgtggtcta

[tRNA+Target1+ gRNA-sc] + [tRNA+Target2+gRNA-sc]:

gaatgattaggcatcgaaccttcaagaatttgattgaataaaacatcttcattcttaagatatgaagataatcttcaaaaggcccctgggaatctgaa
agaagagaagcaggcccatttatatgggaaagaacaatagtatttcttatataggcccatttaagttgaaaacaatcttcaaaagtcccacatcgct
tagataagaaaacgaagctgagtttatatacagctagagtcgaagtagtgattgaacaaagcaccagtggtctagtggtagaatagtaccctgcc
acggtacagacccgggttcgattcccggctggtgcataatggagaacagcatctcggttttagagctagaaatagcaagttaaaataaggctagt
ccgttatcaacttgaaaaagtggcaccgagtcggtgcaacaaagcaccagtggtctagtggtagaatagtaccctgccacggtacagacccgg
gttcgattcccggctggtgcatatcatctatctgtggtctagttttagagctagaaatagcaagttaaaataaggctagtccgttatcaacttgaaaaag
tggcaccgagtcggtgc

Csy4 endonucleases sequence+ GSG linker+P2A:

atggatcattatcttgatattagacttagacctgatccagaatttccaccagctcaacttatgtctgttctttttggaaaacttcatcaagctcttgttgctca
aggaggagatagaattggagtttcttttcctgatcttgatgaatcaagatcaagacttggagaaagacttagaattcatgcttctgctgatgatcttaga
gctttgcttgctagaccttggcttgaaggacttagagatcatcttcaatttggagaaccagctgttgttccacatccaactccttatagacaagtttcaag
agttcaagctaaatctaatccagaaagacttagaagaagacttatgagaagacatgatctttctgaagaagaagctagaaaaagaattcctgata
ctgttgctagagctttggatttgccttttgttacacttagatcacaatctactggacaacattttagactttttattagacatggaccacttcaagttactgctg
aagaaggaggatttacttgttatggactttctaagggaggttttgttccttggtttggatctggagctactaatttttctcttcttaagcaagctggagatgtt
gaagaaaatcctggaccc

CmYLCV+[Csy4+Target1+gRNA-sc]+[Csy4+Target2+gRNA-sc]+Csy4+ T35S(t):

tggcagacatactgtcccacaaatgaagatggaatctgtaaaagaaaacgcgtgaaataatgcgtctgacaaaggttaggtcggctgcctttaat
caataccaaagtggtccctaccacgatggaaaaactgtgcagtcggtttggctttttctgacgaacaaataagattcgtggccgacaggtgggggt
ccaccatgtgaaggcatcttcagactccaataatggagcaatgacgtaagggcttacgaaataagtaagggtagtttgggaaatgtccactcacc
cgtcagtctataaatacttagcccctccctcattgttaagggagcaaaatctcagagagatagtcctagagagagaaagagagcaagtagccta
gaagtagtcaaggcggcgaagtattcaggcacgtggccaggaagaagaaaagccaagacgacgaaaacaggtaagagctaagcttcctgc
aggttcactgccgtataggcagtaatggagaacagcatctcggttttagagctagaaatagcaagttaaaataaggctagtccgttatcaacttgaa
aaagtggcaccgagtcggtgcgttcactgccgtataggcagtatcatctatctgtggtctagttttagagctagaaatagcaagttaaaataaggcta

gtccgttatcaacttgaaaaagtggcaccgagtcggtgcgttcactgccgtataggcaggtcgatcgacaagggtctcacggccatgctagagtcc
gcaaaaatcaccagtctctctctacaaatctatctctctctatttttctccagaataatgtgtgagtagttcccagataagggaattagggttcttataggg
tttcgctcatgtgttgagcatataagaaacccttagtatgtatttgtatttgtaaaatacttctatcaataaaatttctaattcctaaaaccaaaatccagtg
acct

CmYLCV+[tRNA+Target1+gRNA-sc]+[tRNA+Target2-+gRNA-sc]+T35S(t):

Tggcagacatactgtcccacaaatgaagatggaatctgtaaaagaaaacgcgtgaaataatgcgtctgacaaaggttaggtcggctgcctttaat
caataccaaagtggtccctaccacgatggaaaaactgtgcagtcggtttggctttttctgacgaacaaataagattcgtggccgacaggtgggggt
ccaccatgtgaaggcatcttcagactccaataatggagcaatgacgtaagggcttacgaaataagtaagggtagtttgggaaatgtccactcacc
cgtcagtctataaatacttagcccctccctcattgttaagggagcaaaatctcagagagatagtcctagagagagaaagagagcaagtagccta
gaagtagtcaaggcggcgaagtattcaggcacgtggccaggaagaagaaaagccaagacgacgaaaacaggtaagagctaagcttaaca
aagcaccagtggtctagtggtagaatagtaccctgccacggtacagacccgggttcgattcccggctggtgcataatggagaacagcatctcggt
tttagagctagaaatagcaagttaaaataaggctagtccgttatcaacttgaaaaagtggcaccgagtcggtgcaacaaagcaccagtggtctag
tggtagaatagtaccctgccacggtacagacccgggttcgattcccggctggtgcatatcatctatctgtggtctagttttagagctagaaatagcaa
gttaaaataaggctagtccgttatcaacttgaaaaagtggcaccgagtcggtgcttaattaaggtctcaccatggcggccgggagcggccatgcta
gagtccgcaaaaatcaccagtctctctctacaaatctatctctctctatttttctccagaataatgtgtgagtagttcccagataagggaattagggttctt
atagggtttcgctcatgtgttgagcatataagaaacccttagtatgtatttgtatttgtaaaatacttctatcaataaaatttctaattcctaaaaccaaaat
ccagtgacct

Note: Letters marked in red are sequences of sgRNA1 and sgRNA3.
